# Contributions to the knowledge of the Chinese *Primeuchroeus* Linsenmaier, 1968 (Hymenoptera, Chrysididae), with a key to species

**DOI:** 10.3897/zookeys.373.6556

**Published:** 2014-01-23

**Authors:** Na-sen Wei, Paolo Rosa, Zai-fu Xu

**Affiliations:** 1 Department of Entomology, College of Natural Resources and Environment, South China Agricultural University, Guangzhou 510640, P. R. China; 2 Via Belvedere 8/d, I-20881 Bernareggio (MB), Italy

**Keywords:** Chrysididae, *Primeuchroeus*, revision, new record, Oriental, China

## Abstract

The genus *Primeuchroeus* Linsenmaier, 1968 from China is revised and an illustrated identification key is produced for the first time. Three species are recorded from China, with one species, *Primeuchroeus yongdaerianus* Kim, new to China.

## Introduction

*Primeuchroeus* Linsenmaier belongs to the tribe Chrysidini of the subfamily Chrysidinae (Kimsey & Bohart, 1991). Bohart (1988) revised the genus and divided it into five species-groups, with a key to all known species at that time. More detailed diagnosis and discussion of each species-group was published later by Kimsey and Bohart (1991). Linsenmaier (1997) considered three species, *Primeuchroeus alces* Linsenmaier, 1968, *Primeuchroeus paradoxa* Linsenmaier, 1968 and *Primeuchroeus tenuimediata* Linsenmaier, 1968 belonged to the genus *Chrysis*. However, we follow the systematics proposed by Kimsey and Bohart (1991).

Presently 33 species of *Primeuchroeus* are known worldwide (Kimsey and Bohart 1991; Kim 2013), of which 26 are from the Australian region, four are from the Oriental region, one is from the Palaearctic region, one is from both the Oriental and the Palaearctic regions, and one is from the Afrotropical region (Kimsey and Bohart 1991; Madl and Rosa 2012; Kim 2013).

In China, before this study, only two species have been recorded from Taiwan by Tsuneki: *Primeuchroeus crassiceps* (Tsuneki, 1970) and *Primeuchroeus kansitakuanus* (Tsuneki, 1970). In this study, three species are recognized, with one new to China.

## Materials and methods

All specimens were examined and described under stereomicroscope (Olympus SZ61). All photos were taken with a digital camera (CoolSNAP) attached to a Zeiss Stemi 2000-CS stereomicroscope. Images were processed using Image-Pro Plus software.

Morphological terminology mainly follows Kimsey and Bohart (1991).

Abbreviations used in the descriptions as follows: F-I, F-II, F-III, etc. = flagellum I, flagellum II, flagellum III and so on; MOD = midocellar diameter; MS = malar space, the shortest distance between the base of mandible and the margin of compound eye; S-II spots = two oval dark spots on metasomal sternum II; TFC = transverse frontal carina; T-I, T-II, T-III, etc. = metasomal tergum I, tergum II, tergum III and so on.

All specimens are kept in the Hymenopteran Collection, South China Agricultural University, Guangzhou, China (SCAU) and the Shanghai Entomological Museum, Chinese Academy of Science, Shanghai, China (SEM).

## Taxonomy

### Genus Primeuchroeus

Linsenmaier, 1968

http://species-id.net/wiki/Primeuchroeus

Primeuchroeus Linsenmaier 1968: 38. Type species: *Chrysis papuana*Mocsáry 1899. Linsenmaier 1982: 325; Bohart 1988: 21; Kimsey and Bohart 1991: 535; Kim 2013: 95.
Papuachrysis Linsenmaier 1968: 52. Type species: *Chrysis alces*Linsenmaier 1968. Synonymized by Kimsey and Bohart 1991.

#### Diagnosis.

Scapal basin usually with fine transverse striae (Figs 3, 12, 21, 30). TFC often down-curved crescent, sometimes apparently double (Figs 21, 30), rarely flat or absent (Figs 3, 12). Subgenal area defined by carina. Mesopleuron without scrobal sulcus (Figs 6, 15, 24, 33). Forewing with Rs short and ending abruptly (Figs 5, 14, 32), or Rs long and nearly complete (Fig. 23). Lateral margin of T-III edentate (Figs 9, 18, 36), dentate, or convex basally (Fig. 27). Apex of T-III usually round (Figs 7, 16, 34) or obtusely angled medially (Fig. 25), rarely tridentate. Female T-IV with coarse transverse ridges.

#### Key to the Chinese species of *Primeuchroeus* Linsenmaier

**Table:** 

1	Forewing with Rs long, about two times as long as stigma, and bent sharply in the middle (Fig. 23); lateral margin of T-III convex, with a small tooth basally (Fig. 27)	*Primeuchroeus kansitakuanus* (Tsuneki)
–	Forewing with Rs short, about half as long as stigma, and ending obtusely (Figs 5, 14, 32); lateral margin of T-III nearly straight, without tooth basally (Figs 9, 18, 36)	2
2	TFC absent (Figs 3, 12); pronotum with an obtuse angle on each lateral margin (Figs 4, 13)	*Primeuchroeus crassiceps* (Tsuneki)
–	TFC distinct and double (Fig. 30); pronotum with an indistinct angle on each lateral margin (Fig. 31)	*Primeuchroeus yongdaerianus* Kim

### Primeuchroeus crassiceps

(Tsuneki, 1970)

http://species-id.net/wiki/Primeuchroeus_crassiceps

**Figures 1, 2.**
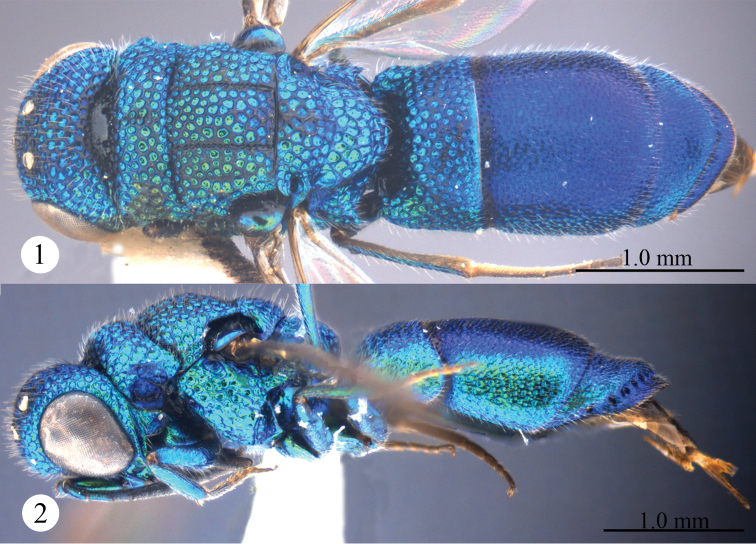
*Primeuchroeus crassiceps* (Tsuneki, 1970), female from Yunnan. **1** Habitus, dorsal view **2** habitus, lateral view.

Figs 1 [Fig F2] [Fig F3] –18

Chrysis crassiceps Tsuneki 1970: 8.
Primeuchroeus crassiceps (Tsuneki, 1970): Bohart 1988: 23; Kimsey and Bohart 1991: 541.

#### Materials.

1♀ (SCAU), Yunnan, Gaoligongshan National Nature Reserve (24°49'N, 98°46'E), 20–21.VII.2006, Jie Zeng, Juan-juan Ma & Bin Xiao leg., No. CP0038; 4♀♀+2♂♂ (SCAU), Yunnan, Gaoligongshan National Nature Reserve, Mailongxia (25°50'23"N, 98°51'23"E), 17.VIII.2005, Kai Wu leg., No. CP0039–0044.

#### Description.

Described after a female from Yunnan. Body length 4.3 mm (Figs 1, 2). Forewing length 3.1 mm. MS = 0.7 MOD. F-I 2.0× as long as wide.

**Figures 3–9. F2:**
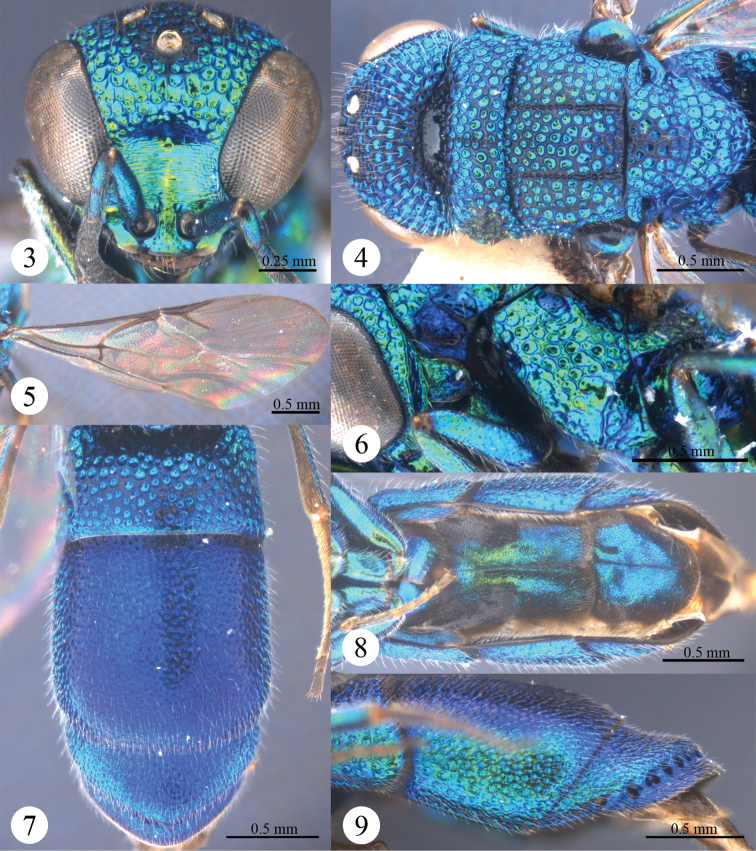
*Primeuchroeus crassiceps* (Tsuneki, 1970), female from Yunnan. **3** Head, anterior view **4** head, pronotum, mesonotum, metanotum, and propodeum, dorsal view **5** forewing **6** pronotum, mesopleuron and metapleuron, lateral view **7** metasoma, dorsal view **8** metasoma, ventral view **9** T-II and T-III, lateral view.

*Head*. Scapal basin deep and impunctate, with transverse striae and a round pit anteromedially (Fig. 3). F-I slightly longer than F-II (Fig. 3). TFC absent (Fig. 3).

*Mesosoma*. Pronotum with anterior declivity polished and impunctate between the two pits (Fig. 4); with an obtuse angle on each lateral margin (Fig. 4), with sublateral carina complete and lateral depression shallow (Fig. 6). Mesonotum with areolate punctures (Fig. 4). Mesopleuron without enlarged foveae along episternal sulcus (Fig. 6), without carina and projection (Fig. 6). Forewing with discoidal cell faint outwardly (Fig. 5); Rs short, 0.6 times as long as stigma, and ending obtusely (Fig. 5). Propodeal angle sharp and pointing backwards (Fig. 4).

*Metasoma*. T-I with sparser and considerably larger punctures than those on T-II and T-III (Fig. 7). S-II spots separated by 1.7 MOD (Fig. 8). T-III slightly bulging before pit row (Fig. 9); apex of T-III round, without transparent rim (Fig. 7); lateral margin of T-III nearly straight, without tooth (Fig. 9).

*Colouration*. Head and mesosoma metallic green, blackish along notauli. Mandible brown, with metallic green basally. Antenna black, with scape, pedicel and basal F-I metallic bluish-green. Tegula metallic bluish-green. Leg metallic bluish-green, with inner surface of tibia and tarsus brown. Metasoma metallic blue, with T-I metallic greenish-blue.

**Figures 10, 11. F3:**
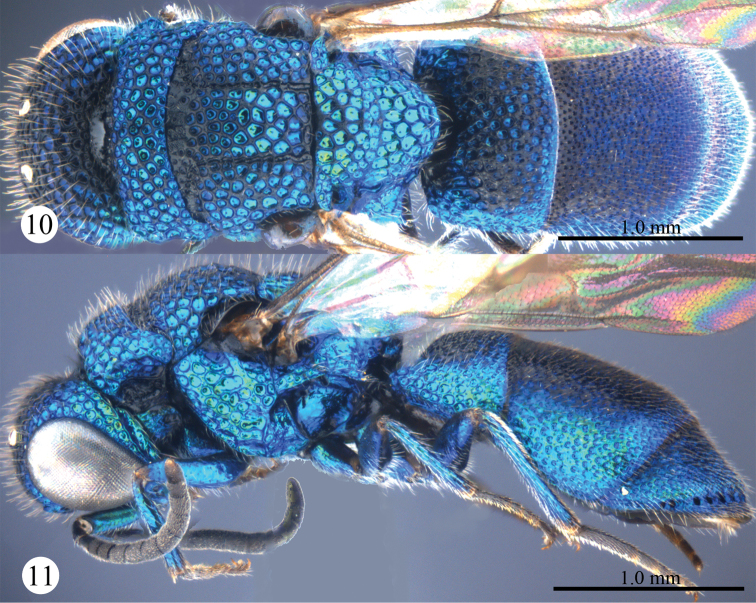
*Primeuchroeus crassiceps* (Tsuneki, 1970), male from Yunnan. **10** Habitus, dorsal view **11** habitus, lateral view.

*Male*. Body length 4.3 mm (Figs 10, 11). Forewing length 3.1 mm. MS = 0.7 MOD. F-I 2.0× as long as wide. Subantennal space = 0.8 MOD. Differing from female as follows: vertex, mesosoma, T-I and T-II darker than those of female; forewing with discoidal cell more distinct than that of female (Fig. 14); apex of T-III rounder than that of female, without angle medially (Fig. 16).

**Figures 12–18.**
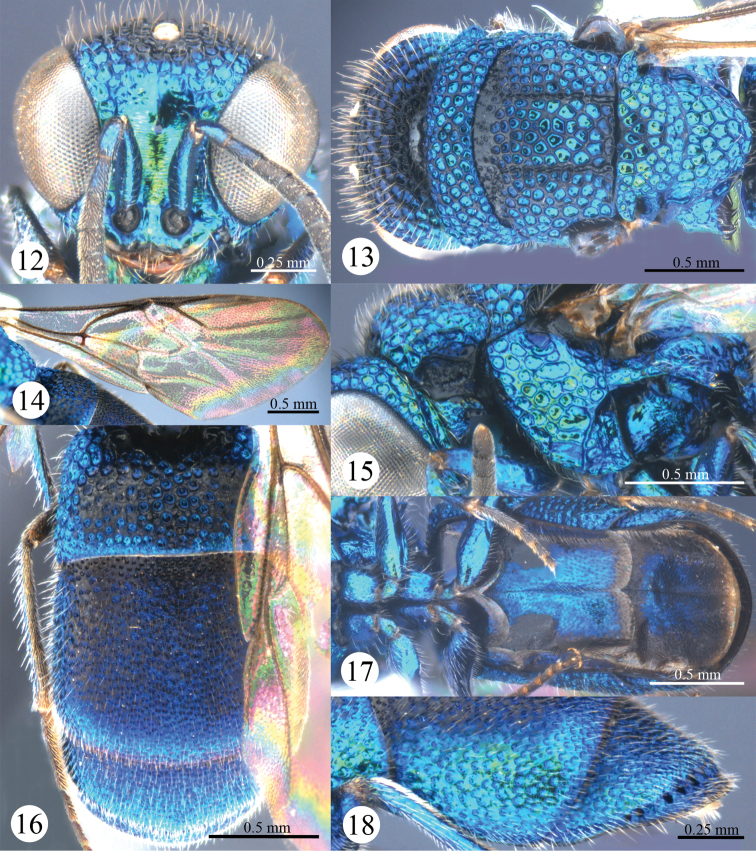
*Primeuchroeus crassiceps* (Tsuneki, 1970), male from Yunnan. **12** Head, anterior view **13** head, pronotum, mesonotum, metanotum, and propodeum, dorsal view **14** forewing **15** pronotum, mesopleuron and metapleuron, lateral view **16** metasoma, dorsal view **17** metasoma, ventral view **18** T-II and T-III, lateral view.

*Variation*. Females (n = 5). Body length 3.5–4.4 mm. Forewing length 3.0–3.4 mm. Rs 0.5–0.6 times as long as stigma. Metasoma with purple tints; F-I subequal to F-II. Males (n = 2). Body length 3.5–4.3 mm. Forewing length 3.0–3.1 mm.

#### Diagnosis.

F-I slightly longer than F-II. TFC absent. Pronotum with an obtuse angle on each lateral margin. Forewing with Rs short, 0.6 times as long as stigma, ending obtusely. Lateral margin of T-III nearly straight, without tooth.

#### Distribution.

China (Taiwan, Yunnan).

#### Biology.

Unknown. Collected in July and August.

#### Remark.

According to Bohart (1988), *Primeuchroeus crassiceps* belongs to the *siamensis* species-group.

### Primeuchroeus kansitakuanus

(Tsuneki, 1970)

http://species-id.net/wiki/Primeuchroeus_kansitakuanus

Figs 19 –27

Chrysis kansitakuanus Tsuneki 1970: 9.
Primeuchroeus kansitakuanus (Tsuneki, 1970): Bohart 1988: 23; Kimsey and Bohart 1991: 542.

#### Materials.

1♀ (SCAU), Zhejiang, Lin’an, Mt. Qingliangfeng (30°04'N, 118°52'E), 9.VIII.2005, Hong-ying Zhang leg., No. 200603255; 1♀ (SEM) Fujian, Da’an (27°51'12.80"N, 117°54'24.42"E), 1.VII.1959, Gen-tao Jin & Yang-ming Lin leg., No. 34022850; 1♀ (SCAU), Hubei, Jingmen, Jingshan (31°1'1'N, 113°78'10"E), 15.VII.2009, Yuan Ye leg., No. CP0029; 13♀♀ (SCAU), Hunan, Mt. Huping, Shinianzigou (29°55'38"N, 118°48'48"E), 9.VII.2009, Ya-li Tang leg., No. CP0002–0014; 4♀♀ (SCAU), Hunan, Mt. Huping, Shinianzigou, 9.VII.2009, Shi-hong Wang leg., No. CP0015–0018; 1♀ (SCAU), Hunan, Mt. Huping, Zongfeng (29°55'N, 118°48'E), 9.VII.2009, Shi-hong Wang leg., No. CP0019; 1♀ (SCAU), Hunan, Mt. Huping, Shuawu village (29°55'N, 118°48'E), 10.VII.2009, Li Ma leg., No. CP0020; 1♀ (SCAU), Hunan, Huaihua (27°33'17"N, 109°59'53"E), VIII.2004, Jian-hua Zhou leg., No. CP0021; 2♀♀ (SCAU), Guangzhou, Wangzishan Forest Park (23°34'49"N, 113°13'21"E), 20.V.2006, Ju-jian Chen & Zai-fu Xu leg., No. CP0030, 0031; 4♀♀ (SCAU), Guangzhou, Liuxihe Forest Park (23°44'31"N, 113°47'0"E), 20.VI.2004, Zai-fu Xu leg., No. CP0032–0035; 2♀♀ (SCAU), Guangdong, Chebaling National Nature Reserve (24°43'N, 114°14'E), 22–28.VII.2008, Zai-fu Xu leg., No. CP0036, 0037; 1♀ (SCAU), Hainan, Mt. Wuzhi (18°51'N, 109°39'E), 15–16.V.2008, Jing-xian Liu leg., No. 200800155; 1♀ (SCAU), Guizhou, Tianzhu (26°54'32"N, 109°12'22"E), VIII.2009, Yang-wen Wang leg., No. CP0001; 2♀♀ (SCAU), Guizhou, Mayang River, Dahe Dam (28°38'12"N, 108°17'13"E), 27.IX–2.X.2007, Jie-min Yao leg., No. CP0027, 0028; 3♀♀ (SCAU), Yunnan, Jinggu, Yunhai Reserve (23°29'37"N, 100°42'39"E), 3.X.2004, Jing-xian Liu leg., No. CP0022–0024; 1♀ (SCAU), Yunnan, Yingjiang, Taiping village (24°39'29"N, 97°51'9"E), 15.VIII.2005, Qiang Li leg., No. CP0025; 1♀ (SCAU), Yunnan, Chenggong, Luoyang (24°55'35'N, 102°48'37"E), 8–19.VIII.2006, Qiang Li leg., No. CP0026.

#### Description.

**Figures 19, 20.**
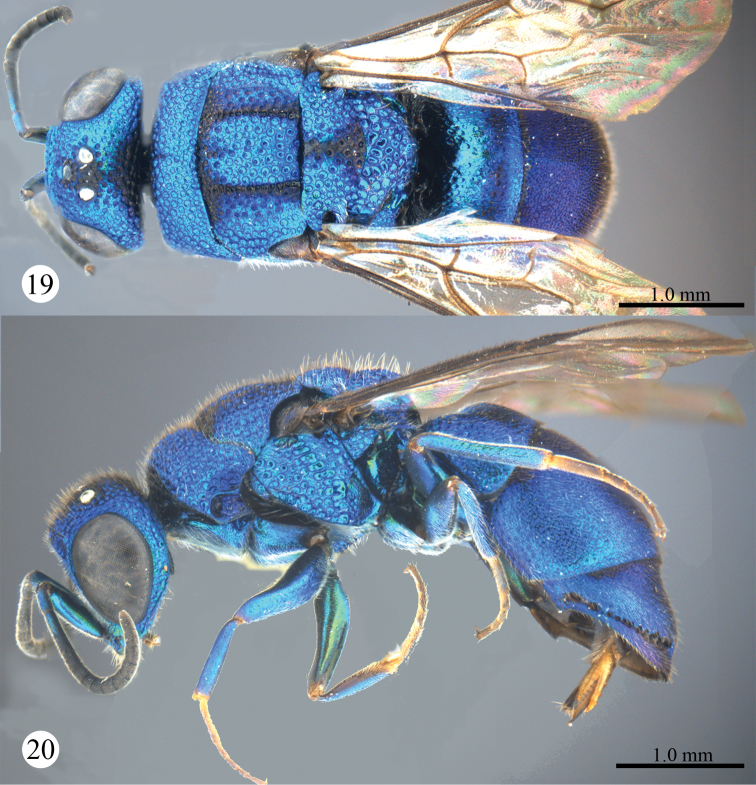
*Primeuchroeus kansitakuanus* (Tsuneki, 1970), female from Guizhou. **19** Habitus, dorsal view **20** habitus, lateral view.

Described after a female from Guizhou. Body length 7.0 mm (Figs 19, 20). Forewing length 4.6 mm. MS = 0.6 MOD. F-I 3.0× as long as wide.

**Figures 21–27.**
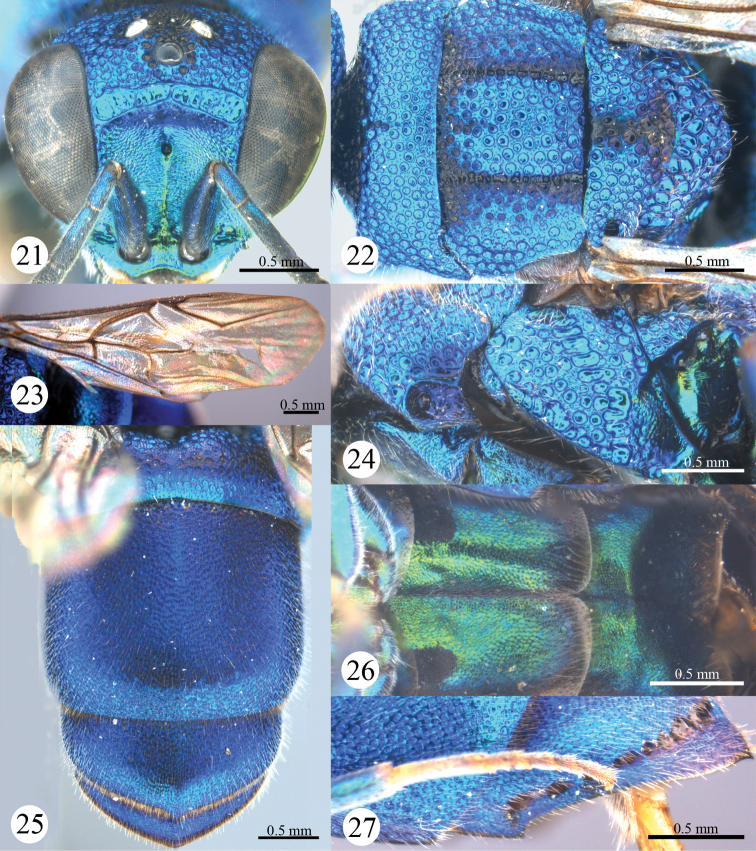
*Primeuchroeus kansitakuanus* (Tsuneki, 1970), female from Guizhou. **21** Head, anterior view **22** pronotum, mesonotum, metanotum, and propodeum, dorsal view **23** forewing **24** pronotum, mesopleuron and metapleuron, lateral view **25** metasoma, dorsal view **26** metasoma, ventral view **27** T-II and T-III, lateral view.

*Head*. Scapal basin deep, with transverse striae and punctures, with a round pit anteromedially (Fig. 21). F-I dinstinctly longer than F-II (Fig. 21). TFC distinct and double (Fig. 21).

*Mesosoma*. Pronotum with anterior declivity polished and impunctate between the two pits (Fig. 22); without angle on each lateral margin (Fig. 22), without sublateral carina, but with lateral depression deep (Fig. 24). Mesonotum with round punctures (Fig. 22). Mesopleuron with enlarged foveae along episternal sulcus (Fig. 24), with scrobal carina and a very small projection near scrobe (Fig. 24). Forewing with discoidal cell distinct (Fig. 23); Rs long, 2.4 times as long as stigma, sharply bent in the middle and nearly complete (Fig. 23). Propodeal angle sharp and pointing backwards.

*Metasoma*. T-I with sparser and considerably larger punctures than those on T-II and T-III (Fig. 25). S-II spots separated by 3.0 MOD (Fig. 26). T-III not bulging before pit row (Fig. 27); apex of T-III obtusely angled medially, without transparent rim (Fig. 25); lateral margin of T-III convex, with a small tooth basally (Fig. 27).

*Colouration*. Face metallic green. Mandible brown, with metallic green basally. Antenna black, with scape, pedicel and basal F-I metallic bluish-green. Vertex and mesosoma metallic bluish-green, with ocellar triangle, anterior part of pronotum, notauli, and metanotum black. Tegula blackish-brown. Leg metallic bluish-green, with tarsus testaceous. Metasoma metallic bluish-green.

*Variation*. Females (n = 40). Body length 5.0–7.0 mm. Forewing length 3.4–4.6 mm. Rs 2.0–2.4 times as long as stigma.

*Male*. Unknown.

#### Diagnosis.

F-I distinctly longer than F-II. TFC distinct and double. Forewing with Rs long, 2.0–2.4 times as long as stigma, and bent sharply in the middle. Lateral margin of T-III convex, with a small tooth basally.

#### Distribution.

China (Zhejiang, Hubei, Hunan, Taiwan, Fujian, Guangdong, Hainan, Guizhou, Yunnan); Vietnam; Malaysia.

#### Biology.

Unknown. Collected from May to October.

#### Remarks.

According to Bohart (1988), *Primeuchroeus kansitakuanus* belongs to the *ghilianii* species-group.

### Primeuchroeus yongdaerianus

Kim, 2013 new to China

http://species-id.net/wiki/Primeuchroeus_yongdaerianus

Figs 28 –36

Primeuchroeus yongdaerianus Kim 2013: 95.

#### Materials.

4♀♀ (SCAU), Yunnan, Gaoligongshan National Nature Reserve (24°49'N, 98°46'E), 20–21.VII.2006, Jie Zeng, Juan-juan Ma & Bin Xiao leg., No. CP0045–0048; 8♀♀ (SCAU), Yunnan, Gaoligongshan National Nature Reserve, Mailongxia (25°50'23"N, 98°51'23"E), 17.VIII.2005, Kai Wu leg., No. CP0049–0056.

#### Description.

**Figures 28, 29.**
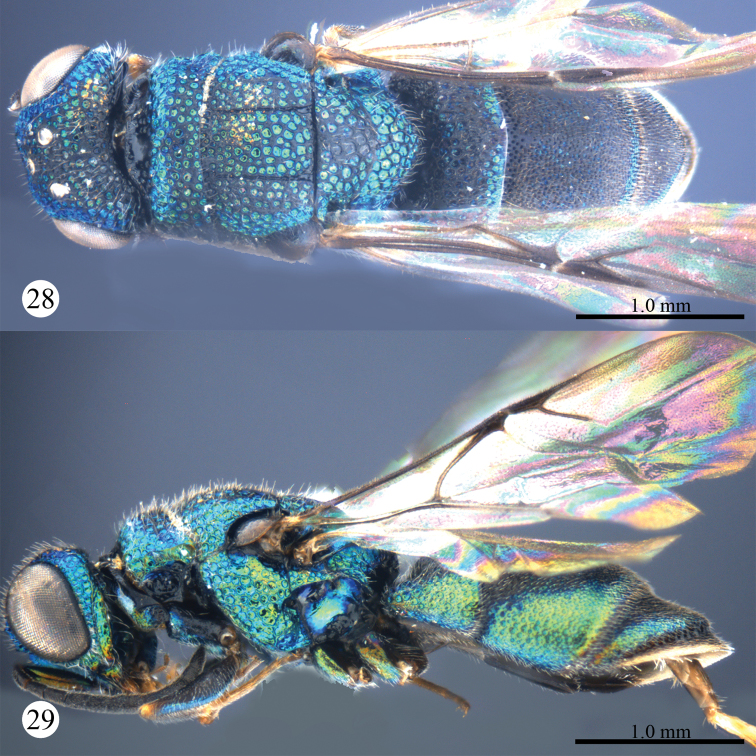
*Primeuchroeus yongdaerianus* Kim, 2013, female from Yunnan. **28** Habitus, dorsal view **29** habitus, lateral view.

Described after a female from Yunnan. Body length 3.1 mm (Figs 28, 29). Forewing length 2.5 mm. MS =1.2 MOD. F-I 2.5× as long as wide.

**Figures 30–36.**
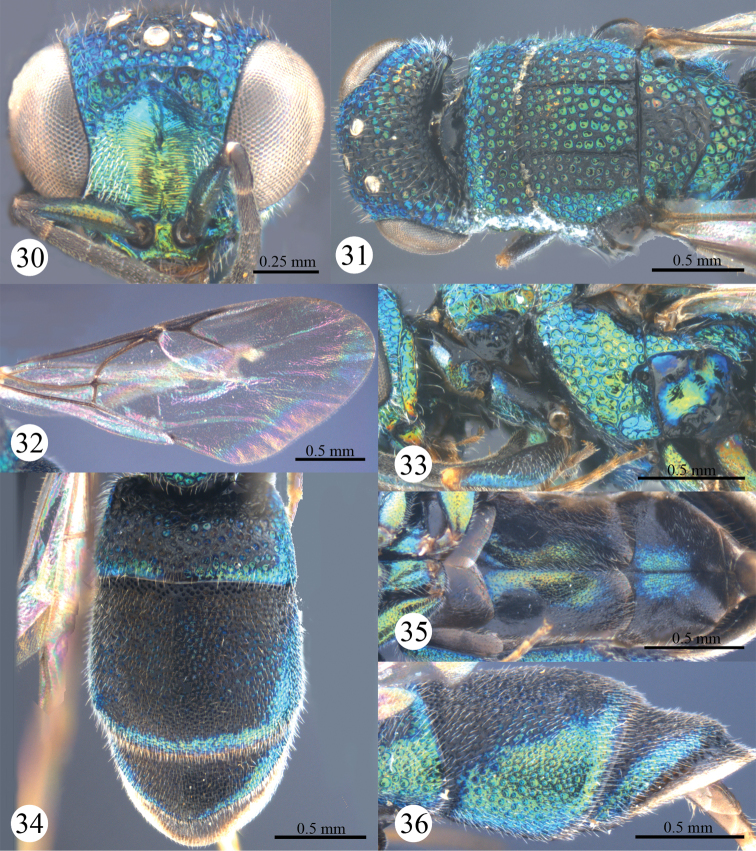
*Primeuchroeus yongdaerianus* Kim, 2013, female from Yunnan. **30** Head, anterior view **31** head, pronotum, mesonotum, metanotum, and propodeum, dorsal view **32** forewing **33** pronotum, mesopleuron and metapleuron, lateral view **34** metasoma, dorsal view **35** metasoma, ventral view **36** T-II and T-III, lateral view.

*Head*. Scapal basin deep and impunctate, with transverse striae and an elongate pit anteromedially (Fig. 30). F-I slightly longer than F-II (Fig. 30). TFC distinct and double (Fig. 30).

*Mesosoma*. Pronotum with anterior declivity polished and impunctate between two pits (Fig. 31); with an indistinct angle on each lateral margin (Fig. 31), with sublateral carina incomplete, and with lateral depression shallow (Fig. 33). Mesonotum with areolate punctures (Fig. 31). Mesopleuron without enlarged foveae along episternal sulcus (Fig. 33), without scrobal carina and projection (Fig. 33). Forewing with discoidal cell faint outwardly (Fig. 32); Rs short, 0.6 times as long as stigma, and ending obtusely (Fig. 32). Propodeal angle sharp and pointing backwards (Fig. 31).

*Metasoma*. T-I with sparser and considerably larger punctures than those on T-II and T-III (Fig. 34). S-II spots separated by 2.3 MOD (Fig. 35). T-III slightly bulging before pit row (Fig. 36); apex of T-III round, with broad testaceous transparent rim (Fig. 34); lateral margin of T-III nearly straight, without tooth (Fig. 36).

*Colouration*. Face metallic green, with yellow reflections. Mandible brown, with metallic green basally. Antenna black, with scape metallic green. Vertex, pronotum, mesonotum, and metanotum metallic bluish-green. with black colour. Tegula blackish-brown, with metallic blue hints. Leg with coxa and femur metallic green; tibia mostly brown, with slight metallic reflections; tarsus brown. Metasoma mostly black, with metallic green reflections posteriorly and laterally on each segment.

*Variation*. Females (n = 12). Body length 2.6–4.1 mm. Forewing length 2.4–3.1 mm. F-I subequal to F-II. Rs 0.6–0.7 times as long as stigma.

*Male*. No available specimens for this study.

#### Diagnosis.

TFC distinct and double. Forewing with Rs short, 0.6 times as long as stigma, and ending obtusely. Lateral margin of T-III nearly straight, without tooth.

#### Distribution.

China (Yunnan); Korea.

#### Biology.

Unknown. Collected in July and August.

#### Remarks.

According to Kim (2013), *Primeuchroeus yongdaerianus* belongs to the siamensis species-group. It is the first record to the Oriental region and to China. The specimens from Yunnan vary from those from Korea by tibia and tarsus brown, and with metallic blue on S-III.

## Supplementary Material

XML Treatment for Primeuchroeus

XML Treatment for Primeuchroeus crassiceps

XML Treatment for Primeuchroeus kansitakuanus

XML Treatment for Primeuchroeus yongdaerianus
